# Radioimmunotherapy consolidation and rituximab maintenance in the initial treatment of follicular lymphoma

**DOI:** 10.1186/2191-219X-1-7

**Published:** 2011-06-20

**Authors:** Franz Buchegger, Oliver W Press

**Affiliations:** 1 Department of Nuclear Medicine, University Hospital of Lausanne, Lausanne, Switzerland; 2 Department of Nuclear Medicine, University Hospitals of Geneva, Geneva, Switzerland; 3 Fred Hutchinson Cancer Research Center, Seattle, WA98109, USA; 4 Division of Oncology, Department of Medicine, University of Washington, Seattle, WA98195, USA

**Keywords:** Radioimmunotherapy consolidation, Rituximab maintenance, Follicular lymphoma

## Abstract

Several reports have documented similar efficacies and tolerable toxicities of radioimmunotherapy (RIT) consolidation and rituximab maintenance after initial R-chemotherapy of follicular lymphoma. The relative merits of these two interventions are currently under discussion. We now raise the question whether both RIT consolidation and rituximab maintenance should be used together aiming to augment the results achievable with R-chemotherapy.

## Letter to the Editor

The recent review of T.M. Illidge on radioimmunotherapy (RIT) of lymphoma highlighted the inherent potential of this particular treatment [1]. While convinced of the efficacy of RIT he regretted the low implementation of RIT in current clinical practice.

We would like to elaborate further on the biological agents that have shown efficacy in treatment of follicular lymphoma. In a small series of relapsed indolent lymphoma patients treated in Switzerland with 131I-tositumomab (Bexxar^®^, GlaxoSmithKline, Brentford, UK), we experienced several long-lasting complete remissions with six of 12 patients (50%) reaching 10-years progression free survival without any further treatment [2]. Similar 10-year progression-free survivals after ^131^I-tositumomab in relapse have been reported by another group, though at a somewhat lower rate [3]. The Southwest Oncology Group (SWOG) has demonstrated that 67% of follicular lymphoma patients remained progression free more than 5 years after consolidation of CHOP chemotherapy with ^131^I-tositumomab [4]. Yet, another group has communicated a 50% complete response (CR) rate after 10 years following an initial treatment using abbreviated fludarabine combined with ^131^I-tositumomab [5]. A high number of persistent CRs at 5 to 6 years was also reported for ^131^I-tositumomab alone in the initial treatment of follicular lymphoma [6].

High efficacy of RIT with 90Y-ibritumomab has also been reported repeatedly including a report demonstrating 5-year relapse free survival in about 20% of relapsed patients [7]; however, 10-year observations are currently not available to us.

^90^Y-ibritumomab (Zevalin^®^, Spectrum Pharmaceuticals, Henderson, NV, USA) is the only RIT currently approved in Europe, and its successful use in consolidation treatment following chemotherapy has been well documented [8]. On the other hand, rituximab (Mabthera^®^, Rituxan^®^, Roche Ltd., Genentech, Basel, Switzerland) maintenance treatment after R-chemotherapy was recently shown to improve the relapse-free survival in a large phase III study [9].

Several reports have documented similar efficacies of RIT consolidation and rituximab maintenance, though these approaches have not been formally tested in a randomized trial. Both complementary treatments have moderate and/or transient side effects. The mode of action of these two added therapies, however, are different. Rituximab maintenance is an immunotherapy while ^131^I-tositumomab and ^90^Y-ibritumomab are targeted radiation therapies administered in combination with two injections of moderate amounts of unlabeled antibody. The latter approach is supported by long-standing evidence showing that radiotherapy may occasionally be curative when used as initial treatment for localized follicular lymphoma [10]. We envision a similar potential for RIT given in advanced disease either alone or combined with chemotherapy, as initial treatment or in relapse [2,3,5].

The 10-year progression-free survival as observed after ^131^I-tositumomab either alone or combined with chemotherapy, upfront or at relapse of follicular lymphoma, appears to be an important milestone. It is anticipated that this approach might afford a low recurrence rate in subsequent years, analogous to that observed after external beam radiotherapy [10]. The low-energy electrons emitted by ^131^I are also prone to eradicate microscopic disease, as has been shown by others and us in RIT of small-sized lung or liver metastatic disease, respectively [11,12].

Further improved biological efficacy in NHL might be achieved by combining anti-CD20 rituximab treatment with other antibodies directed against other antigens, such as anti-CD22 or anti-CD40, utilizing humanized antibodies, or novel anti-CD20 antibodies with modified Fc domains providing increased affinity for Fc receptors and improved effector functions as discussed previously [13,14].

In current practice, rituximab has appropriately assumed a dominant position in treatment of lymphoma both in combination with chemotherapy and as maintenance [9]. We now raise the question whether both rituximab and RIT should be used together as complementary methods to augment the results achievable with chemotherapy and whether this combined modality approach might afford additive or synergistic benefit. This strategy might also allow reducing chemotherapy as has been shown with abbreviated fludarabine combined with RIT [5]. An attenuated R-CHOP as recently described for elderly patients could possibly be envisaged in such a triple therapy approach [15].

We acknowledge that there is currently little published data demonstrating the efficacy of anti-CD20 RIT following rituximab-containing induction chemotherapy regimens. This information gap will be partially remedied by a phase II study investigating this combined approach that has recently been piloted by SWOG in the NCT00770224 trial, which is assessing the toxicity and efficacy of R-CHOP induction chemotherapy followed by ^131^I-tositumomab consolidation and 4 years of rituximab maintenance. This study will assess the potential impact of administering rituximab anti-CD20 antibody with CHOP prior to anti-CD20 RIT [16], though in this trial rituximab was deliberately omitted from the last two cycles of CHOP chemotherapy, to minimize blocking of CD20 antigenic sites prior to RIT. If favorable results are achieved in this phase II trial, a phase III randomized study comparing this "triple" approach with maintenance rituximab alone or consolidation RIT alone following induction with R-chemotherapy would be warranted.

## List of abbreviations

*RIT*: radioimmunotherapy; *R-chemotherapy *:rituximab and chemotherapy; *R-CHOP*: combined rituximab, cyclophosphamide, doxorubicin, vincristine, and prednisone; *SWOG *(US): Southwest Oncology Group.

## Competing interests

OWP declares a compensated consultant and advisory role and having received honoraria from Hoffmann-LaRoche/Genentech and Spectrum Pharmaceuticals as well as having received research funding from Hoffmann-LaRoche/Genentech. FB has no competing interest to declare.

## Authors' contributions

Both authors express in this letter their opinion, edited and corrected this letter and approved its final version.
